# Who Creates Criminal Children?

**Published:** 1893-10-14

**Authors:** 


### A Physiological Lantern.

### WHO CREATES CRIMINAL CHILDREN?

If we may judge from the behaviour of many parents, we must conclude that those parents believe that their children have created themselves, and have voluntarily endowed themselves with all their unpleasant and sometimes most exasperating peculiarities. But it is obvious that no child—not even Topsy, who "spected she had growed"—ever yet performed the miraculous feat of "making itself." This is worthy of consideration. If a child has a squint or bandy legs, we may be sorry for him, or even annoyed at the fact, but we do not box the poor little fellows ears every time he squints or makes awkward attempts to beat his better-legged brother at running. We recognise that he was born so, and that he must have had ancestors, near or remote, who have squinted or had a rachitic constitution. Moreover, if we are at all sensible, we let him have the help of an oculist for his squint, and of an orthopædist for his bandy legs.

But if the little man has been born with a fiendish temper, or with a tendency to lying, or to theft, what do we do then? The present writer once saw a huge blacksmith take a thick cane in both hands, and, commanding his son of eight or nine years old to take off his jacket, beat him on the unclothed back and shoulders until the child could hardly stand. The boy had stolen a pencil from one of his school companions. The sight of the huge man of six feet thundering blow upon blow down upon the little trembling and terrified child was one of horror. The smith himself was one of the most selfish brutes in the small parish, and the child was only indulging prematurely the greedy desire for the possession of portable property which he had inherited from his father. The boy died of consumption not long afterwards.

It ought to be recognised that a bad temper, or a tendency to lying, or theft, or any other vicious peculiarity, is in many cases congenital. In other words, the child who is born with any of these peculiarities is no more responsible for their presence in his blood and brain than he is for his squint or his bandy legs. But, exclaims the unreasoning parent, or the half-taught village magistrate, are we not to deal with the little brat; is he to be allowed to lie and rob the hen-roost or the orchard at his pleasure? By all means deal with him, but according to reason, and not according to uncultured and heathenish brutality. If the oculist, for example, were to adopt your principles, he would not try to cure the squint by a careful surgical operation, but he would forthwith knock out the patient's eye. Similarly the orthopædist would not by patient manipulation, or the excision of a piece of bone, try to make his "bandy" patient a pair of straight and respectable legs; he would knock off the offending members at the knee, and bid the victim march, like a disabled Jack Tar, on his stumps.

The rough and ready methods of the old school, we insist, will not do in morals any more than they will do in surgery. Nay, inasmuch as matters mental and moral are much finer and more delicate than matters physical, there is more necessity for careful and competent handling in the former than in the latter. The first feeling in the mind of a really instructed person who sees a young child endowed with falsehood, or with undue greed, or with a fiendish temper, is one of pity—it is not one of blame. Such a person no more thinks of blaming the hapless child for his inherited falseness of character than he does for his inherited squint or his inherited bandy legs.

What, then, is to be done with congenital moral and mental defects? They are to be treated exactly as congenital defects of a physical kind are treated—that is, scientifically. Now, the surgeon treats squint or bandy legs without passion. Anger in him we feel would be absurd. That he should scold or thrash his bandy-legged patient would be to behave like a madman. What he does is to set to work in calm reason, in cold blood, not to make the bandy-legged boy perfect as to his legs, but to bring the legs as nearly to the normal standard of straightness as possible. He is content with that—he expects no more. Similarly the passionate boy, the liar, the cheat, are to be treated calmly, in cold blood. They are to be taught; not merely told, but taught; taught patiently, taught slowly, taught day by day for years. But what then? Are they never to be punished, never to be thrashed? As a matter of teaching and discipline they may be punished and thrashed; but in anger and hot blood, as if they deserved nothing but indignation, they should never be thrashed, or punished in any way. But if teaching and mild discipline will not cure them, what then? Why, then, it is certain that brutality will not cure them; and it must be recognised that, like a proportion of the cases of squint or bandy legs, they are incurable.

What should be our feelings towards children and young people who are manifestly and incurably criminal? Our feelings should be exactly the same as we indulge towards those who suffer from incurable physical ills—the same, but even more tender. Look back a little upon the personal and family history of the congenital criminal. He came into the world by no choice of his own—he could not choose his parents—if he could have done, it is certain he would not have selected that brutal father, that vicious and self-willed mother. Perhaps his grandfathers and grandmothers on both sides have been of a low, ignorant, and brutal type. What a concentration of hereditary ignorance, stupidity, cunning, and vice has met in that poor boy's brain and blood! He is as naturally born to crime as the fox is to the love of poultry, or the snake to the use of his poison fangs when threatened with danger.

It is to be definitely recognised and admitted that certain persons are congenitally criminal, as others are congenitally bandy-legged or congenitally insane. We claim for the congenital criminal as kindly treatment as we claim for the insane and the physically defective. This is the only logical position which scientific persons can take up. The thing can be put into as convincing a syllogism as the most rigid logician can desire. "All persons born with injurious incurable peculiarities ought to be pitied—some congenital criminals are persons born with incurable peculiarities, therefore some congenital criminals ought to be always treated from the standpoint of pity."

For practical purposes there ought to be a class of medical experts whose duty it should be to deal with the cases of apparently incurable criminals. Such criminals, when it has been scientifically decided that they are incurable, should be recognised as patients in hospitals or asylums. Their family history and their personal history should be carefully inquired into, and they should be put under a course of rational moral medicine at the expense of the State. In short, the habitual criminal's prison should be a hospital-school. In due time a certain number of congenital criminals would be so far improved that, like the boy with the cured bandy legs, they would be able to walk, on the whole, morally straight. Such as these should, at the proper time, be set at liberty to take care of themselves, on the understanding that they would be free to return to the hospital-school if they found the criminal tendency too strong for them. A certain proportion of congenital criminals would be found to be permanently incurable. These would have to be confined to the school-hospital for the term of their natural life. Their lives should in that case be made as happy for themselves, and as profitable to the State, as possible —seeing that, though they are vicious and regardless of other people's rights, they are so by virtue of hereditary forces over which they have had no control, and which have proved too strong for them.

